# Accuracy of Artificial Intelligence for Cervical Vertebral Maturation Assessment—A Systematic Review

**DOI:** 10.3390/jcm13144047

**Published:** 2024-07-10

**Authors:** Wojciech Kazimierczak, Maciej Jedliński, Julien Issa, Natalia Kazimierczak, Joanna Janiszewska-Olszowska, Marta Dyszkiewicz-Konwińska, Ingrid Różyło-Kalinowska, Zbigniew Serafin, Kaan Orhan

**Affiliations:** 1 Department of Radiology and Diagnostic Imaging, Collegium Medicum, Nicolaus Copernicus University in Torun, Jagiellońska 13-15, 85-067 Bydgoszcz, Poland; 2 Kazimierczak Private Medical Practice, Dworcowa 13/u6a, 85-009 Bydgoszcz, Poland; 3 Department of Interdisciplinary Dentistry, Pomeranian Medical University in Szczecin, 70-111 Szczecin, Poland; 4 Chair of Practical Clinical Dentistry, Department of Diagnostics, Poznań University of Medical Sciences, 61-701 Poznań, Poland; 5 Department of Dental and Maxillofacial Radiodiagnostics, Medical University of Lublin, 20-093 Lublin, Poland; 6 Department of Dentomaxillofacial Radiology, Faculty of Dentistry, Ankara University, Ankara 06500, Turkey; 7 Medical Design Application and Research Center (MEDITAM), Ankara University, Ankara 06500, Turkey; 8 Department of Oral Diagnostics, Faculty of Dentistry, Semmelweis University, 1088 Budapest, Hungary

**Keywords:** artificial intelligence (AI), lateral cephalogram, cervical vertebrae, machine learning, cervical vertebral maturation assessment, skeletal maturity

## Abstract

**Background/Objectives:** To systematically review and summarize the existing scientific evidence on the diagnostic performance of artificial intelligence (AI) in assessing cervical vertebral maturation (CVM). This review aimed to evaluate the accuracy and reliability of AI algorithms in comparison to those of experienced clinicians. **Methods:** Comprehensive searches were conducted across multiple databases, including PubMed, Scopus, Web of Science, and Embase, using a combination of Boolean operators and MeSH terms. The inclusion criteria were cross-sectional studies with neural network research, reporting diagnostic accuracy, and involving human subjects. Data extraction and quality assessment were performed independently by two reviewers, with a third reviewer resolving any disagreements. The Quality Assessment of Diagnostic Accuracy Studies (QUADAS)-2 tool was used for bias assessment. **Results:** Eighteen studies met the inclusion criteria, predominantly employing supervised learning techniques, especially convolutional neural networks (CNNs). The diagnostic accuracy of AI models for CVM assessment varied widely, ranging from 57% to 95%. The factors influencing accuracy included the type of AI model, training data, and study methods. Geographic concentration and variability in the experience of radiograph readers also impacted the results. **Conclusions:** AI has considerable potential for enhancing the accuracy and reliability of CVM assessments in orthodontics. However, the variability in AI performance and the limited number of high-quality studies suggest the need for further research.

## 1. Introduction

In the last few years, there has been an increase in the amount of scientific evidence supporting the diagnostic accuracy and effectiveness of AI in various clinical scenarios [1]. Due to the nature of diagnostic imaging and its repetitive analysis of specific image features, radiology is an area of medicine in which AI is developing most rapidly [2]. Owing to its significant use of imaging and emphasis on cephalometric analysis, orthodontics is particularly well suited for the implementation of AI [3]. Recently, the effectiveness of AI has been evaluated in a number of utilizations associated with orthodontic treatment, including automated landmark detection and cephalometric analysis, dental and temporomandibular joint (TMJ) diagnostics, treatment planning, treatment outcome evaluation, patient monitoring, and skeletal age assessment [4]. The results of scientific research indicate that AI can significantly enhance the efficiency of clinical orthodontic practice and diminish the workload of practitioners [5,6]. However, the impact of AI algorithms on patient care remains a matter of rising concern.

Growth and maturation are critical factors in the field of orthodontics because they are closely linked to the effectiveness of orthodontic treatment. Patients treated with orthodontic appliances tend to achieve optimal growth and develop a harmonious relationship in the masticatory system before attaining skeletal maturity [7]. The growth rate and facial development stage are vital for lasting orthodontic results. Precise assessment of these factors is necessary to minimize undesired post-treatment changes due to ongoing facial growth [8]. Previous studies have shown that properly aligning orthodontic treatment with a patient’s growth phases can increase its effectiveness [9,10].

Adolescent growth rates vary significantly; therefore, chronological age alone does not sufficiently predict the extent of remaining growth [11,12]. The use of skeletal age is a widely accepted and reliable method for evaluating individual growth, and it can be determined through two main approaches: cervical vertebrae maturation (CVM) and wrist X-rays [9,13,14,15,16]. Both growth intensity and growth potential are important factors in terms of proper treatment timing or optimal choice of the treatment strategy. Since the standard diagnostic orthodontic routine does not involve the use of wrist X-rays due to additional radiation exposure, currently, the method of choice in skeletal maturity assessment in these patients remains CVM [17]. CVM utilizes lateral cephalograms frequently acquired during treatment planning and has already shown accuracy and reliability in skeletal age assessment [16,18].

It was further modified by Hassel and Farman in 1995 [19] and Bacetti in 2005 [20]. The method involves evaluating the development and fusion of the cervical vertebrae, particularly the morphology of the second, third, and fourth vertebrae. Since its introduction, the method has been widely utilized in orthodontics to help determine the optimal timing for orthodontic treatment and for monitoring skeletal growth [16]. However, this method requires additional training and experience, and some studies have shown its poor reproducibility, particularly in classifying the shapes of C3 and C4 vertebral bodies [21,22]. Since AI has already shown its ability to detect features that may be hidden to human readers [23,24], its incorporation in CVM assessment may aid clinicians in proper diagnosis. Due to the continuously increasing number of research papers, it was pertinent to conduct a systematic review of the current body of literature.

The present systematic review aimed to identify and summarize the existing scientific evidence concerning the diagnostic performance of AI in CVM assessment

## 2. Materials and Methods

### 2.1. Search Strategy and Eligibility Criteria

This systematic review was conducted according to the PRISMA (Preferred Reporting Items for Systematic Reviews and Meta-Analyses) statement [25], Supplementary Material Tables S1 and S2 and the guidelines from the Cochrane Handbook for Systematic Reviews of Interventions [26]. On 16 January 2024, a series of preliminary searches of the following databases were performed: PubMed, PMC, Scopus, Web of Science, Embase, and the Dental & Oral Health Source EBSCO. The final search proceeded on 31 January 2024 using all of the abovementioned search engines. The combination of different Boolean operators AND/OR and MeSH/non-MeSH terms was used to select appropriate studies: [artificial intelligence] OR [deep learning] OR [automated] OR [machine learning] AND [cervical vertebral maturation] OR [skeletal maturity]. Additional studies were selected by searching the reference lists of all included articles, and all related papers were also screened through the PubMed database. The final search string included the following terms: (“cervical vertebrae” OR “cervical vertebra”) AND (“maturation” OR “CVM” OR “CVMS” OR “skeletal age” OR “skeletal maturation” OR “skeletal development”) AND (“deep learning” OR “machine learning” OR “CNN” OR “SVM” OR “decision tree” OR “random forest” OR “convolutional neural network” OR “neural network” OR “Bayesian” OR “artificial intelligence”). EndNote 21 software was used to collect references and remove duplicates. Study selection was independently carried out by two reviewers (WK and MJ) and evaluated through Cohen’s kappa coefficient; any disagreements were resolved by a third expert reviewer (JJO). The same two reviewers extracted study characteristics, such as authors, year of publication, algorithm architecture, dataset partition (training and test), and algorithm accuracy metrics. Based on PICO(S) [27], the framework of this systematic review was developed as follows: population: orthodontic patients; comparison: evaluation of the maturation stage of cervical vertebrae according to the assessment of artificial intelligence software and experienced clinicians; outcomes: accuracy of cervical vertebrae assessment according to CVM or CVMS; and studies: cross-sectional studies with neural network research. The included articles discussed the clinical efficiency of neural networks for evaluating cervical vertebral maturation.

Studies were included if they met the following criteria: (1) cross-sectional studies with neural network research for cervical vertebral maturation assessment, (2) studies reporting diagnostic accuracy, (3) human studies, (4) studies with a sample size of at least 30, and (5) studies published in peer-reviewed journals.

The exclusion criteria were as follows: (a) conference papers, (b) case reports, (c) descriptions of technique, (d) research without quantitative evaluation, (e) book chapters, and (f) records unrelated to the topic of the review. No language restrictions were applied.

After the results were retrieved from the search engines to create a database, duplicates were removed. Then, the titles and abstracts were independently analyzed by two authors (WK and NK) following the inclusion criteria. Full-text articles of potentially eligible studies were then retrieved and reviewed for final inclusion. Disagreements were resolved by discussion with the third author (JJO) by creating a working spreadsheet to verify the results by the Cochrane Collaboration guidelines [26]. Cohen’s K coefficient for the agreement between the authors indicates perfect agreement between the authors and was equal to 0.98.

### 2.2. Data Extraction and Quality Assessment

Data on study characteristics, such as study design, sample size, AI algorithm used, CVM method used, and accuracy measures, were extracted using a standardized data extraction form. The quality of the included studies was assessed using the Quality Assessment of Diagnostic Accuracy Studies (QUADAS)-2 tool. The tool includes four domains: patient selection, index test, reference standard, and flow and timing. Each domain is evaluated for bias risk, and the first three domains are also evaluated for applicability concerns. The use of signaling questions aids in assessing bias. QUADAS-2 is used in four steps: summarizing the review question, tailoring the tool to provide review-specific guidelines, creating a primary study flow diagram, and evaluating bias and applicability. It enhances the transparency of bias and applicability ratings in primary diagnostic accuracy studies [28].

## 3. Results

### 3.1. Search Results

**Figure 1:**
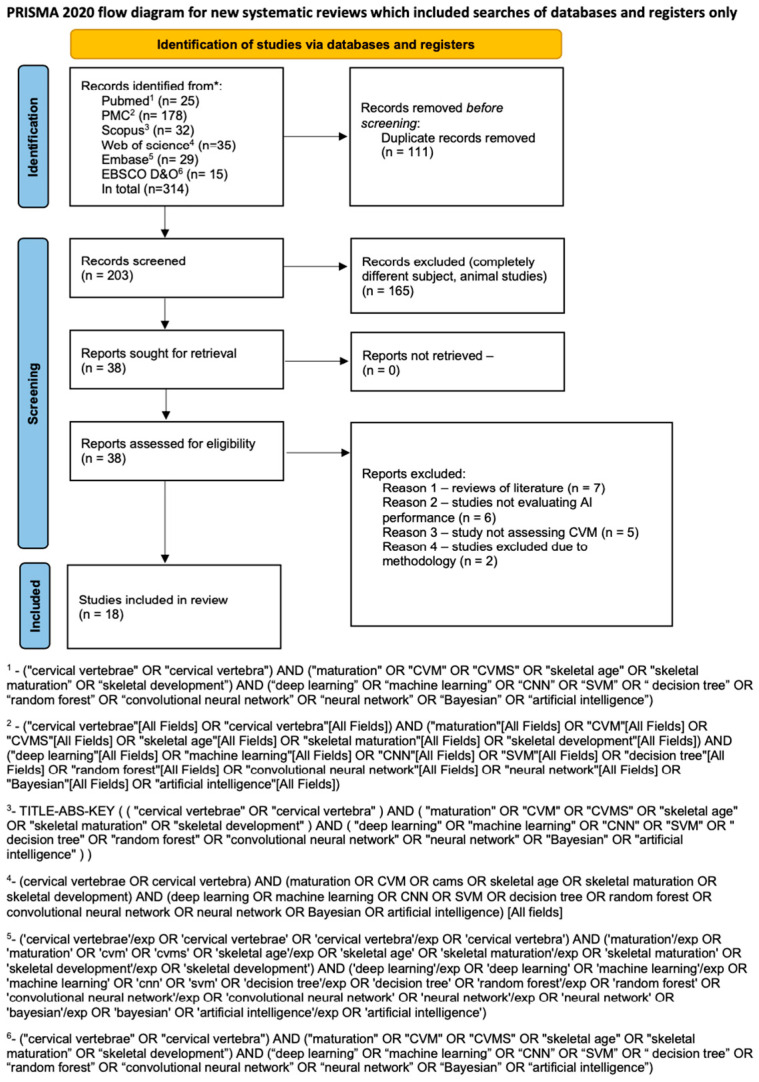
Prisma 2020 flow diagram (*—name of the database).

An initial search using tailored queries to each database resulted in a total of 314 articles. After 111 duplicate articles were removed, the remaining 203 studies were initially screened. Subsequently, 165 studies were removed because they were out of the scope of the review. Figure 1 presents Prisma flow diagram thoroughly describing the search process. Both reviewers had a high level of agreement in this phase, achieving a Cohen’s kappa of 0.98. Few disagreements were resolved by a third reviewer (JJO). Subsequently, 38 articles underwent full-text screening, of which twenty were excluded because seven were reviews of the literature, six did not evaluate AI systems, five did not evaluate cervical maturation, and two did not present a structured methodology with clear results (Supplementary Material Table S3). Ultimately, 18 articles were found to be eligible for inclusion in the review. The data obtained from the studies are presented in Table 1.

The studies were predominantly conducted in Turkey (*n* = 7), followed by Korea and China (*n* = 3 each), with additional studies from the USA (*n* = 2), Iran (*n* = 2), and France (*n* = 1). Notably, the eighteen included studies were from only twelve research groups. This indicates the niche nature of the study and the fact that it is being developed by a small group of researchers throughout the world. The overall sample size included in the review was 30,275 cephalograms. The number of samples varied from 419 to 10,200 among the studies.

### 3.2. Risk of Bias

The overall risk of bias in the studies included in the review was rather low or unclear. However, there are some studies that provided proper descriptions of the methods applied. Two studies were at high risk of bias. The main shortcomings in patient selection are the lack of a detailed description of subject enrollment and the randomization of the subjects before manual analysis of radiographs, which could have resulted in bias. If the study provided accurate patient demographics and vertebral maturity assessments for the patients included in the study, the risk of bias was considered low. If the study did not provide complete demographic data or an assessment of vertebral maturity was not indicated, the risk of bias was considered unclear. If the study only stated that a certain number of radiographs were included in the study, without providing their characteristics, the risk was considered high. For the same reasons, it remains unclear whether the results presented in studies can be applied to a wider spectrum of populations studied. One study should have indicated that the risk of bias was high, as the authors did not indicate any characteristics of the included radiographs beyond their number. The risk of index test bias was considered low if both intra-rater and inter-rater compliance were examined. If one piece of information about one of these examinations was missing, the risk was described as unclear. Thus, if an error study was not performed in any of the trials, the points were not determined manually by more than one orthodontist. Due to the prevalence and validity of the vertebral evaluation method, the risk of bias due to the reference standard was low, except for the study by Seo et al. [45], who did not describe the method of reference. All but one study clearly described the intervals and timing. The applicability concerns regarding patient selection remains the same due to the nature of the study material. In the case of Makaremi et al. [42], applicability of index test is unclear, as both detailed description and timing of index test are lacking, while by Seo et al. [46] the description of index test and reference standard left too many uncertainties, therefore the risk should be considered high. The summary of risk of bias assessment is presented in Table 2.

### 3.3. Methods of CVM Assessment and Reference Standards

The reference standards were set according to three methods. Most of the studies have used the method by Bacetti et al. [30,31,33,34,39,43,44,45,46], followed by the methods by Hassel and Farman [29,36,37,38] and by McNamara and Franchi [32,35,39,41,42]. The number of observers evaluating radiographs, their experience and their professions varied widely among the studies. One of the Seo et al. studies did not mention the number of readers [45]. In eight of the studies, there was only one reader [32,34,36,37,38,41,43,44]. However, in studies by Kök et al., the reader assessed the images twice with a fixed time interval [36,37,38]. Among the remaining studies, the number of readers was greater—up to four—in the case of the study by Amasya et al. [31].

### 3.4. AI Models

Various AI models were employed in the included studies to assess CVM stages. Predominantly, 14 studies have utilized supervised learning techniques, with convolutional neural networks (CNNs) being a common choice [29,33,34,35,39,40,41,43,44,45,46], often deployed in both pretrained and newly trained forms. Other AI models tested include artificial neural networks (ANNs) [30,31,36,38], decision trees [30,36], logistic regression random forests [30], support vector machines [30,36], and clinical decision support systems (CDSSs) [31]. Some studies have explored unsupervised learning and novel approaches such as label distribution learning [32].

### 3.5. Diagnostic Accuracy

**Figure 2:**
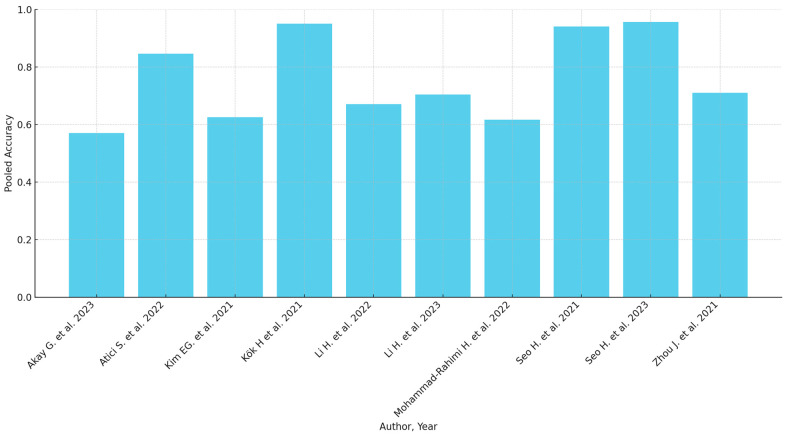
Pooled accuracy of the AI models (when available) [29,32,35,37,39,40,42,44,45,46].

**Figure 3:**
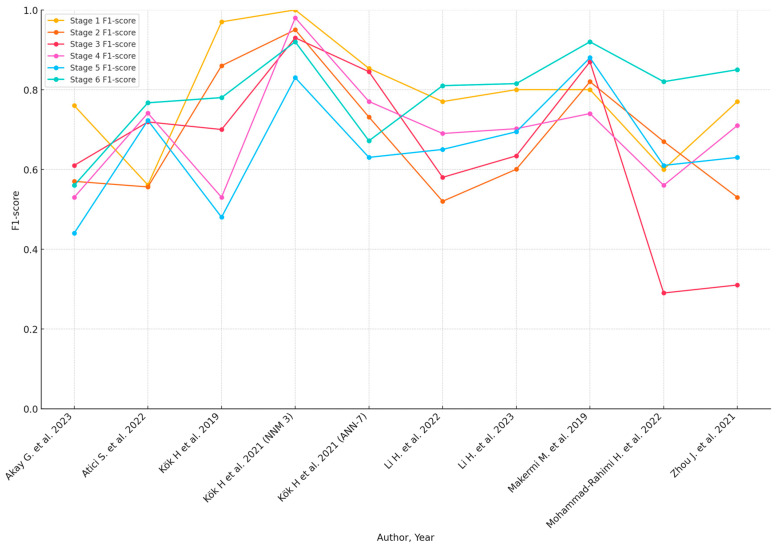
F1-Scores of AI models for each CVM stage (when available) [30,33,37,38,40,41,42,43,47].

Subgroup analyses based on geographic location, sample size, and AI model type highlighted variations in diagnostic accuracy. The pooled accuracy varied from 0.57 (Akay et al. [29]) to 0.956 (Seo et al. [45]). Sensitivity analyses confirmed the robustness of the findings, with predominantly consistent results across different study designs and populations. However, studies with greater methodological rigor and larger sample sizes tended to report more reliable diagnostic performance. A summary of the diagnostic accuracy metrics presented in the included studies can be found in Table 3. When available, the detailed accuracy metrics of each maturation stage are included in the Table 3. Graphical presentations of the available accuracy metrics are presented in Figure 2 and Figure 3.

Seo, Atici and Kim have presented results of calculations as a confusion matrix [33,35,44,45]. The findings of the four studies were not included in the table [30,31,34,43]. Amasya et al. presented only the results of calculations of concordance between human expert readers and selected AI systems [30,31]. Radwan et al. assessed three sets of stages: prepubertal (stages 1 and 2), pubertal (3 and 4), and postpubertal (5 and 6) [43]. Khazaei et al. assessed the accuracy of the model in three- and two-class scenarios [34].

## 4. Discussion

Human maturation is a continuous process, making the estimation of CVM challenging, with approximately one in three cases misclassified [47]. This is typically converted into a classification problem by discretizing continuous CVM levels into six classes, posing challenges in achieving satisfactory performance even for experienced radiologists. However, AI has shown promising results in various dental fields by enhancing human performance and accelerating decision-making processes [48]. While AI has demonstrated superior diagnostic accuracy in skeletal age assessment using wrist and index finger X-rays [49,50], its accuracy in the estimation of CVM remains variable. Although there are already two systematic reviews regarding the use of AI in CVM assessment [51,52], a constantly expanding body of literature provides additional original articles that need to be systematically reviewed. Rana et al. included 13 papers [51], whereas Mathew included eight papers [52]. Thus, it was necessary to systematically review the current body of literature. This systematic review aims to map the existing scientific evidence on the diagnostic performance of AI in CVM assessment, with a focus on the diagnostic accuracy and operational characteristics of various AI approaches.

The 18 studies included in this review demonstrated a wide range of pooled diagnostic accuracies, from 57% to over 95%, highlighting both the potential and limitations of AI technologies in CVM assessment. These variations can be attributed to several factors, including the choice of AI models, the nature of the training data, and the methods employed in each study. Most studies have utilized convolutional neural networks (CNNs) [29,33,34,35,39,40,41,43,44,45,46], reflecting the prevailing trend toward employing deep learning techniques for complex image recognition tasks in medical diagnostics. The performance of these CNN models often surpasses that of traditional methods, particularly when pretrained models are adapted for specific tasks [53]. This adaptation likely benefits from transfer learning, where a model developed for one task is repurposed for another related task, bringing in preexisting knowledge that can be fine-tuned with a smaller set of targeted data. However, the integration of AI into clinical practice raises significant concerns about the generalizability of these models. Most studies were geographically concentrated in countries such as Turkey [29,30,31,36,37,38,43], Korea [35,44,45], and China [39,40,46], which may influence the diversity of training datasets. Such datasets may not adequately represent the global population, potentially limiting the applicability of these AI models in different demographic settings. Moreover, the reliance on data from specific research groups further narrows the diversity of data, potentially leading to models that perform well on specific types of data but fail to generalize across broader populations.

The methodological approaches used to assess the performance of AI models varied across the studies. Some studies employed cross-validation [29,32,35] techniques to mitigate overfitting and enhance the ability of models to generalize to new data. However, the lack of uniformity in validation methods, such as the variation in the number of folds used in cross-validation [37,46], introduces inconsistencies in assessing model performance. Additionally, the review revealed a high degree of variability in the experience and number of readers evaluating the radiographs, ranging from single reader [32,36,41,43,44] assessments to multiple readers with assessments at different intervals. This variability could introduce additional biases into the training data, as the interpretation of CVM stages is subject to inter-rater and intra-rater variability. The results of some of the studies were also affected by the lower number of stages assessed (prepubertal, pubertal, and postpubertal) [34,43]. Furthermore, the ethical considerations of deploying AI in clinical settings were not adequately addressed in all studies, ensuring the transparency of AI processes, ethical data collection, and maintaining patient confidentiality, which was reflected by the majority (12 out of 18 studies) of the studies scoring unclear to high for patient selection in the risk of bias assessment using the QUADAS-2 tool.

**Figure 4:**
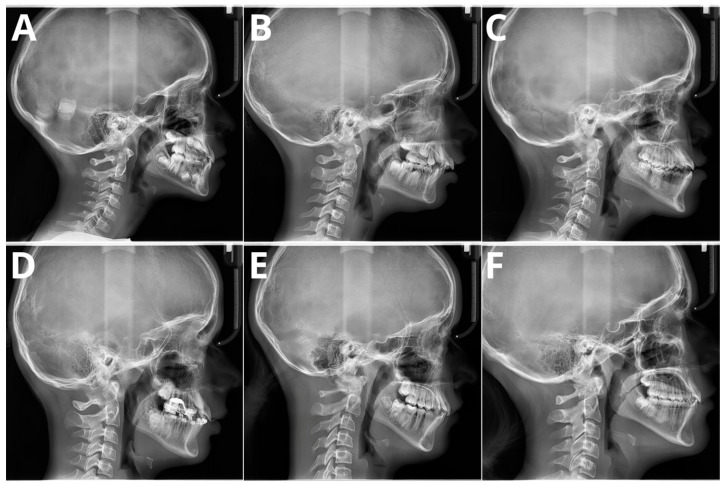
Six maturation stages according to the method by Bacetti et al. (**A**) stage 1, 7 years old female; (**B**) stage 2, 9 years old female; (**C**) stage 3, 11 years old male; (**D**) stage 4, 12 years old male; (**E**) stage 5, 13 years old female; (**F**) stage 6, 13 years old female. Population of single, private orthodontic center, rater—orthodontist with 11 years of experience.

The significant problem associated with CVM evaluation is high inter- and intra-rater variability. A recent paper by Shoretsaniti et al. [54] evaluated the reproducibility and efficiency of CVM assessment. The study included evaluations by six experts in radiology and orthodontics. The intra-rater reliability ranged from 77.0% to 87.3%, meaning that up to 1/4 of the diagnoses of CVM stage were changed. The results of the inter-rater agreement were even worse, with an absolute agreement calculated at 42.8%. The study also showed the lowest reproducibility for stage 3, a crucial stage that marks the beginning of pubertal growth. These results align with other studies that show significant discrepancies in CVM assessment [22,55,56]. Such low scores of both inter- and intra-rater reproducibility indicate that the assessment of CVM stage is biased due to high variability among raters. Therefore, the results of studies showing more than 90% AI accuracy in CVM assessment should be considered very optimistic. It should be emphasized that individual errors and inconsistencies by raters assessing the CVM stage in the training sample significantly impact the learning process of the applied AI model. However, as stated in a Nature paper by Topol [57], AI will likely boost human performance and accelerate decision-making in currently problematic tasks. Figure 4 presents samples of all six stages verified according to the method by Bacetti et al. [20].

With the increased use and popularity of cone beam computed tomography (CBCT) in orthodontic treatment planning, future studies could test the efficacy of AI in assessing CVM using CBCT data. Given the availability and widespread use of CBCT, incorporating this technology could also help reduce multiple radiation exposures. However, to date, there are no studies published on this topic. Additionally, an interesting direction could be the use of MRI in CVM assessment, potentially leading to a radiation-free method of skeletal age assessment. Furthermore, future research should focus on testing AI models on more diverse sample sizes to decrease bias. Since most of the studies evaluated in the present systematic review were conducted in Asia, it is uncertain whether the findings can be generalized to other and more diverse populations. Collaboration among researchers is essential to achieve these goals and enhance the robustness of AI models in clinical applications.

A recent paper by Obuchowski et al. [58] critically evaluated and proposed an appropriate research protocol for multireader-multicase (MRMC) studies. Due to the rapid development of AI and the necessity of assessing the diagnostic accuracy of tested AI models, MRMC study design continues to play a key role in the translation of novel imaging tools to clinical practice. Unlike most medical studies, MRMC requires a reference standard and sampling from both reader and patient populations, making these studies costly and time-consuming. The authors indicated that investigators often attempt numerous analyses and report only the most promising results. Moreover, evaluations based on a single reader’s opinion are highly subjective and can significantly affect model performance metrics, resulting in overly enthusiastic reports. Therefore, the required number of readers, preferably from different institutions and with varying levels of expertise, should be at least five [58,59]. None of the studies provided such a high number of expert readers, with a predominance of one- or two-reader studies. In addition to this significant variability in CVM stage assessment [47], we believe that despite initial optimistic results, the technology of AI-CVM assessment still requires extensive research before it can be routinely applied in clinical practice. However, given these highly encouraging results, we anticipate that future advancements in AI technology will improve the diagnostic accuracy of CVM tools, potentially making them as reliable as wrist X-ray assessments for determining skeletal maturity.

This study has several limitations, including significant heterogeneity among the included studies in terms of study design, sample size, and the AI algorithms used. These variations could impact the generalizability and comparability of the findings.

## 5. Conclusions

Despite the promising results, the studies exhibited heterogeneity in the AI algorithms used, sample sizes, and study designs, which could influence the generalizability of the findings. The risk of bias was generally low, although some studies showed unclear risk, mainly due to the lack of detailed methodological descriptions.

In conclusion, AI has considerable potential for enhancing the accuracy and reliability of CVM assessments in orthodontics. The pooled accuracy for CVM stage assessment varied from 0.57 to 0.956. However, the variability in AI performance and the limited number of high-quality studies suggest the need for further research.

## Figures and Tables

**Table 1:** Characteristics of the included studies.

Study No	Author, Year	Country	Sample Size; (Training/Test Ratio) [%]	Tested AI Model	Reference Standard	CVM Method Used	Outcome
1	Akay G. et al., 2023 [29]	Turkey	588; (60/40)	SL; CNNs, newly trained models	Two radiologists	Hassel-Farman	As a result of training that lasted 40 epochs, 58% training and 57% test accuracy were obtained. The model obtained results that were very close to the training on the test data. On the other hand, it was determined that the model showed the highest success in terms of precision and F1-score in CVM Stage 1 and the highest success in recall value in CVM Stage 2.
2	Amasya et al., 2020 [30]	Turkey	647; (80/20)	ANN, decision tree, logistic regression, RF, and SVM	Two experts	Bacetti et al.	The results of interobserver agreement assessment between AI and ANN showed CVM stage classifier models with substantial to almost perfect agreement (weighted kappa 0.76–0.92).
3	Amasya et al., 2020 [31]	Turkey	647 + 72 (90/10)	Clinical decision support system (CDSS), ANN	Four observers	Bacetti et al.	Intraobserver agreement ranges were as follows: weighted kappa (wk) 5 0.92–0.98, Cohen’s kappa (ck) 5 0.65–0.85, and 70.8–87.5%. Interobserver agreement ranges were as follows: wk 5 0.76–0.92, ck 5 0.4–0.65, and 50–72.2%. Agreement between the ANN model and observers 1, 2, 3, and 4 were as follows: wk 5 0.85 (ck 5 0.52, 59.7%), wk 5 0.8 (ck 5 0.4, 50%), wk 5 0.87 (ck 5 0.55, 62.5%), and wk 5 0.91 (ck 5 0.53, 61.1%), respectively (*p* = 0.001). An average of 58.3% agreement was observed between the ANN model and the human observers.
4	Atici S. et al., 2022 [32]	USA	1018; (70/30)	Unsupervised learning; Label distribution learning, DL; newly trained model	One orthodontist	McNamara, Franchi & Bacetti	The proposed CNN model preceded with a layer of tunable directional filters achieved a validation accuracy of 84.63% in CVM stage classification into five classes, exceeding the accuracy achieved with the other DL models investigated. The custom-designed CNN method also achieved 75.11% in six-class CVM stage classification. The effectiveness of the directional filters is reflected in the improved performance attained in the results.
5	Atici S. et al., 2023 [33]	USA	1018; (80/20)	SL; CNNs, newly trained models	Two orthodontists	Bacetti et al.	The proposed innovative model which uses a parallel structured network preceded with a preprocessing layer of edge enhancement filters achieved a validation accuracy of 82.35% in CVM stage classification on female subjects, and 75.0% in CVM stage classification on male subjects, exceeding the accuracy achieved with the other DL models investigated. The effectiveness of the directional filters is reflected in the improved performance attained in the results. If AggregateNet is used without directional filters, the test accuracy decreases to 80.0% on female subjects and to 74.03% on male subjects.
6	Khazaei M. et al., [34]	Iran	1846; (80/20)	SL; CNNs, newly trained models	One orthodontist, twice in one-month interval	Bacetti et al.	The CNN based on the ConvNeXtBase-296 architecture had the highest accuracy for automatically assessing pubertal growth spurts based on CVM staging in both three-class (82% accuracy) and two-class (93% accuracy) scenarios. Given the limited amount of data available for training the target networks for most of the architectures in use, transfer learning improves predictive performance.
7	Kim E.G. et al., 2021 [35]	Korea	600; (80/20)	SL; CNNs, pretrained and newly trained models	Two specialists	McNamara & Franchi	The combination of the CNN with a region-of-interest detector and segment or module was significantly more accurate (62.5%) than without them.
8	Kök H. et al., 2019 [36]	Turkey	300; (80/20)	SL; k-NN, NB, decision tree, ANN, SVM, RF and logistic regression; pretrained models	One orthodontist, twice in one-month interval	Hassel & Farman	ANN had the second highest and most stable accuracy values in CVM assessment (stages 1–4, 6–68, 8–93%) except CVS5 (47, 4%).
9	Kök H. et al., 2021 [37]	Turkey	360; (80/20 and 70/30)	SL; NNM and NBM; newly trained models	One orthodontist, twice at 15-day interval	Hassel & Farman	The highest determination success rate was obtained in NNM 3 (0.95) and the lowest in NBM 4 (0.50). The determination success of NBM 1 and NBM 3 was almost similar (0.60). The success of NNM 2 did not differ much from that of NNM 1 (0.94). The determination success of stage 5 was relatively lower than the others in NNM 1 and NNM 2 (0.83). The NNMs were more successful than the NBMs in our developed models. It is important to determine the effective ratio and/or measurements that will be useful for differentiation.
10	Kök H. et al., 2021 [38]	Turkey	419; (70/30)	SL; ANN; newly trained models	One orthodontist, twice at 20-day interval	Hassel & Farman	Significantly positive correlations between hand-wrist maturation level, CVS and ages. ANN-7 model accuracy value was 0.9427. The highest model accuracy of 0.8687 with least linear measurements was obtained by drawing 13 linear measurements, using vertical measurements and indents. The growth development periods and gender were determined from CVM using ANN successfully.
11	Li H. et al., 2022 [39]	China	6079; (70/30)	SL; CNNs, newly trained models	Two orthodontists	McNamara	The final classification accuracy ranking was ResNet152 > DenseNet161 > GoogLeNet > VGG16, as evaluated on the test set. ResNet152 proved to be the best model among the four models for CVM classification with a weighted κ of 0.826, an average AUC of 0.933 and total accuracy of 67.06%. The F1 score rank for each subgroup was: CS6 > CS1 > CS4 > CS5 > CS3 > CS2. The areas of the third (C3) and fourth (C4) cervical vertebrae were activated when CNNs were assessing the images.
12	Li H. et al., 2023 [40]	China	10,200; (70/30)	SL; CNNs, newly trained models	Three orthodontists	Bacetti et al.	The system has achieved good performance for CVM assessment with an average AUC (the area under the curve) of 0.94 and total accuracy of 70.42%, as evaluated on the test set. The Cohen’s kappa between the system and the expert panel is 0.645. The weighted kappa between the system and the expert panel is 0.844. The overall ICC between the psc-CVM assessment system and the expert panel was 0.946. The F1 score rank for the psc-CVM assessment system was: CVS (cervical vertebral maturation stage) 6 > CVS1 > CVS4 > CVS5 > CVS3 > CVS2.
13	Makermi M. et al., 2019 [41]	France	1870; (80/20)	SL; CNN; pretrained and newly trained models	One radiographer	McNamara & Franchi	The results show the performances of the proposed method with different numbers of images for training, evaluation and testing and different preprocessing of the datasets. The highest accuracy (0.967–1.0) was achieved with 1870 images used for training and entropic filtering.
14	Mohammad-Rahimi H. et al., 2022 [42]	Iran	890; (70/30)	SL; Transfer learning models; pretrained and newly trained for two datasets.	Two orthodontists	McNamara & Franchi	ResNet101 showed best performance. Six-class CVM diagnosis in ResNet101 model showed validation and test accuracy of 62.63% and 61.62%, respectively. With three-class classification, the model’s validation and test accuracy were 75.76% and 82.83%, respectively.
15	Radwan M.T. et al., 2019 [43]	Turkey	1501; (80/20)	SL; CNNs, newly trained models	One orthodontist	Bacetti et al. (3 stages)	The ICC was valued at 0.973, weighted Cohen’s kappa standard error was 0.870 ± 0.027 which shows high reliability of the observers and excellent level of agreement between them, the segmentation network achieved a global accuracy of 0.99 and the average dice score over all images was 0.93. The classification network achieved an accuracy of 0.802, class sensitivity of (prepubertal 0.78; pubertal 0.45; postpubertal 0.98), respectively, per class specificity of (prepubertal 0.94; pubertal 0.94; postpubertal 0.75), respectively.
16	Seo H. et al., 2021 [44]	Korea	600; (80/20)	SL; CNNs, pretrained and newly trained models	One radiologist	Bacetti et al.	Of all the tested AI models, a pretrained network, Inception-ResNet-v2, had the highest accuracy of 0.941. It also had the highest recall and precision scores among all pretrained models tested.
17	Seo H. et al., 2023 [45]	Korea	600; (80/20)	SL; CNNs, newly trained models	Not mentioned	Bacetti et al.	All deep learning models demonstrated more than 90% accuracy, with Inception-ResNet-v2 performing the best, relatively. In addition, visualizing each deep learning model using Grad CAM led to a primary focus on the cervical vertebrae and surrounding structures.
18	Zhou J. et al., 2021 [46]	China	1080; (90/10)	SL; CNNs, newly trained models	Two examiners; disagreements resolved by third expert	Bacetti et al.	In general, the agreement between AI results and the gold standard was good, with the intraclass correlation coefficient (ICC) value being up to 98%. Moreover, the accuracy of CVM staging was 71%. In terms of F1 score, CS6 stage (85%) ranked the highest accuracy.

Abbreviations: SL—supervised learning; NBM—naive Bayes model; ANN—artificial neural network; SVM—support vector machine; RF—random forest; k-NN—k-nearest neighbor, NB—naive Bayes; NNM—artificial neural network model.

**Table 2:** Risk of bias assessment according to the QUADAS-2 tool.

Authors/Year	Risk of Bias				Applicability Concerns		
	Patient Selection	Index Test	Reference Standard	Flow and Timing	Patient Selection	Index Test	Reference Standard
Akay G. et al., 2023 [29]	Unclear	Low	Low	Low	Unclear	Low	Low
Amasya et al., 2020 [31]	Unclear	Low	Low	Low	Unclear	Low	Low
Amasya et al., 2020 [30]	Unclear	Low	Low	Low	Unclear	Low	Low
Atici S. et al., 2022 [32]	Low	Low	Low	Low	Low	Low	Low
Atici S. et al., 2023 [33]	Low	Low	Low	Low	Low	Low	Low
Khazaei M et al., 2023 [34]	Unclear	Unclear	Low	Low	Unclear	Low	Low
Kim E.G. et al., 2021 [35]	Unclear	Low	Low	Low	Unclear	Low	Low
Kök H. et al., 2019 [36]	Unclear	Unclear	Low	Low	Unclear	Low	Low
Kök H. et al., 2021 [37]	Low	Unclear	Low	Low	Low	Low	Low
Kök H. et al., 2021 [38]	Unclear	Unclear	Low	Low	Unclear	Low	Low
Li H. et al., 2022 [39]	Unclear	Unclear	Low	Low	Unclear	Low	Low
Li H. et al., 2023 [40]	Low	Unclear	Low	Low	Low	Low	Low
Makermi M. et al., 2019 [41]	High	Unclear	Low	Unclear	High	Unclear	Low
Mohammad-Rahimi H. et al., 2022 [42]	Unclear	Low	Low	Low	Unclear	Low	Low
Radwan M.T. et al., 2019 [43]	Low	Low	Low	Low	Low	Low	Low
Seo H. et al., 2021 [44]	Low	Unclear	Low	Low	Low	Low	Low
Seo H. et al., 2023 [45]	Unclear	Unclear	High	Low	Unclear	High	Low
Zhou J. et al., 2021 [46]	Unclear	Low	Low	Low	Unclear	Low	Low

**Table 3:** Comparison of the diagnostic accuracy parameters of the best-performing AI models.

Study No	Author, Year	Tested AI Model	Stage 1	Stage 2	Stage 3	Stage 4	Stage 5	Stage 6	Pooled Accuracy
1	Akay G. et al., 2023 [29]	CNN (40 epochs)	Precision 0.82; Recall 0.7; F1-score 0.76	Precision 0.47; Recall 0.74; F1-score 0.57	Precision 0.64; Recall 0.58; F1-score 0.61	Precision 0.52; Recall 0.54; F1-score 0.53	Precision 0.55; Recall 0.37; F1-score 0.44	Precision 0.52; Recall 0.60; F1-score 0.56	0.57
2	Atici S. et al., 2022 [32]	CNN, images prefiltered	Precision 0.599; Recall 0.528; F1-score 0.561	Precision 0.55; Recall 0.562; F1-score 0.556	Precision 0.671; Recall 0.774; F1-score 0.719	Precision 0.724; Recall 0.758; F1-score 0.741	Precision 0.765; Recall 0.685; F1-score 0.723	Precision 0.789; Recall 0.747; F1-score 0.767	0.8463
3	Atici S. et al., 2023 [33]	AggregateNet with a set of tunable directional edge enhancers, CNN model							Female 0.824, Male 0.75
4	Kim EG. et al., 2021 [35]	Model-3, CNN							0.625
5.	Kök H. et al., 2019 [36]	Decision tree	Accuracy 0.97; Precision 0.93; Recall 0.97; F1-score 0.97	Accuracy 0.96; Precision 0.89; Recall 0.83; F1-score 0.86	Accuracy 0.9; Precision 0.68; Recall 0.71; F1-score 0.7	Accuracy 0.85; Precision 0.55; Recall 0.51; F1-score 0.53	Accuracy 0.87; Precision 0.47; Recall 0.5; F1-score 0.48	Accuracy 0.91; Precision 0.78; Recall 0.78; F1-score 0.78	NA
6	Kök H. et al., 2021 [37]	NNM 3 (70–30%)	Precision 1.0; Recall 1.0; F1-score 1.0	Precision 0.95; Recall 0.95; F1-score 0.95	Precision 0.93; Recall 0.93; F1-score 0.93	Precision 0.95; Recall 1.0; F1-score 0.98	Precision 0.83; Recall 0.83; F1-score 0.83	Precision 0.95; Recall 0.90; F1-score 0.92	0.95
7	Kök H. et al., 2021 [37]	ANN-7 model	Specificity 0.954; Sensitivity (Recall) 0.914; F1-score 0.8533	Specificity 0.957; Sensitivity (Recall) 0.7; F1-score 0.7313	Specificity 0.9628; Sensitivity (Recall) 0.8695; F1-score 0.845	Specificity 0.9628; Sensitivity (Recall) 0.7428; F1-score 0.7703	Specificity 0.9140; Sensitivity (Recall) 0.6571; F1-score 0.6301	Specificity 0.9512; Sensitivity (Recall) 0.6285; F1-score 0.6717	0.9427
8	Li H. et al., 2022 [39]	ResNet152	Precision 0.74; Recall 0.79; F1-score 0.77	Precision 0.52; Recall 0.52; F1-score 0.52	Precision 0.59; Recall 0.56; F1-score 0.58	Precision 0.73; Recall 0.66; F1-score 0.69	Precision 0.66; Recall 0.64; F1-score 0.65	Precision 0.77; Recall 0.84; F1-score 0.81	0.6706
9	Li H. et al., 2023 [40]	Psc-CVM	Precision 0.8559; Recall 0.7509; F1-score 0.8000	Precision 0.5704; Recall 0.6335; F1-score 0.6003	Precision 0.6067; Recall 0.6639; F1-score 0.6340	Precision 0.7510; Recall 0.6592; F1-score 0.7021	Precision 0.6760; Recall 0.7137; F1-score 0.6943	Precision 0.8185; Recall 0.8117; F1-score 0.8151	0.704
10	Makermi M. et al., 2019 [41]	NN, 900 images, 7 layers	Accuracy 0.93; Precision 0.99; Recall 0.67; F1-score 0.8	Accuracy 0.939; Precision 0.94; Recall 0.73; F1-score 0.82	Accuracy 0.952; Precision 0.94; Recall 0.81; F1-score 0.87	Accuracy 0.924; Precision 0.59; Recall 0.99; F1-score 0.74	Accuracy 0.966; Precision 0.84; Recall 0.93; F1-score 0.88	Accuracy 0.969; Precision 0.97; Recall 0.88; F1-score 0.92	NA
11	Mohammad-Rahimi H. et al., 2022 [42]	ResNet-101 (test set)	Precision 0.6; Recall 0.6; F1-score 0.6	Precision 0.64; Recall 0.70; F1-score 0.67	Precision 0.25; Recall 0.33; F1-score 0.29	Precision 0.52; Recall 0.60; F1-score 0.56	Precision 0.67; Recall 0.57; F1-score 0.61	Precision 0.88; Recall 0.78; F1-score 0.82	0.6162
12	Seo H. et al., 2021 [44]	Inception-ResNet-v2							0.941
13	Seo H. et al., 2023 [45]	Inception-ResNet-v2							0.956
14	Zhou J. et al., 2021 [46]	CNN	Precision 0.67; Recall 0.92; F1-score 0.77	Precision 1.0; Recall 0.36; F1-score 0.53	Precision 0.25; Recall 0.4; F1-score 0.31	Precision 0.83; Recall 0.63; F1-score 0.71	Precision 0.46; Recall 1.0; F1-score 0.63	Precision 1.0; Recall 0.74; F1-score 0.85	0.71

## Data Availability

Data are available from the corresponding author upon reasonable request.

## Supplementary Materials

The following supporting information can be downloaded at: https://www.mdpi.com/article/10.3390/jcm13144047/s1, Table S1: Prisma 2020 Checklist, Table S2: Prisma 2020 for Abstract Checklist, Table S3: Studies excluded after full-text analysis [12,16,51,60,61,62,63,64,65,66,67,68,69,70,71,72,73,74,75,76].
