# Supplementary material for: Accuracy of Artificial Intelligence for Cervical Vertebral Maturation Assessment—A Systematic Review
Source: J Clin Med. 2024 Jul 10;13(14):4047. doi: 10.3390/jcm13144047 (PMC11277636; doi:10.3390/jcm13144047)
Supplement: Supplementary file 1 [file jcm-13-04047-s001.zip › Table S3.pdf]

Table S3. Studies excluded after full-text analysis

Title	Year	Journal	Authors	Search engine	Language	Reason of exclusion
Use of artificial intelligence in determination of bone age of the healthy individuals: A scoping review [1]	2023	Journal of the World Federation of Orthodontists	Adeel Ahmed Bajjad et al.	Scopus	English	review
CURRENT NEURAL NETWORKS DEMONSTRATE POTENTIAL IN AUTOMATED CERVICAL VERTEBRAL MATURATION STAGE CLASSIFICATION BASED ON LATERAL CEPHALOGRAMS [2]	2024	Journal of Evidence-Based Dental Practice	Cao Lingyun et al.	Scopus	English	review
Cervical Vertebral Maturation Assessment using various Machine Learning techniques on Lateral cephalogram: A systematic literature review [3]	2023	Journal of Oral Biology and Craniofacial Research	Rana Shailendra Singh	PMC	English	review
Machine Learning and Orthodontics, Current Trends and the Future Opportunities: A Scoping Review [4]	2021	American Journal of Orthodontics and Dentofacial Orthopedics	Mohammad Rahimi Hossein et al.	Scopus	English	review
Neural networks for classification of cervical vertebrae maturation: a systematic review	2022	Angle Orthodonics	Reji Mathew et al.	PMC	English	review
Artificial Intelligence (AI)-Based Systems for Automatic Skeletal Maturity Assessment through Bone and Teeth Analysis: A Revolution in the Radiological Workflow? [5]	2023	Applied Sciences	Caloro Elena et al.	PMC	English	review
Is the cervical vertebral maturation (CVM) method effective enough to replace the hand-wrist maturation (HWM) method in determining skeletal maturation?-A systematic review. [6]	2018	Eureopean Journal of Radiology	Szemraj Agnieszka et al.	PMC	English	review
Machine learning for image analysis in the cervical spine: Systematic review of the available models and methods [7]	2022	BRAIN AND SPINE	Goedmakers C. et al.	Scopus	English	review

Utilization of an Artificial Intelligence Program Using the Greulich-Pyle Method to Evaluate Bone Age in the Skeletal Maturation Stage [8]	2023	DBpia	Kim Jihoon et al.	PMC	Korean	methodology
Development of a multi-stage model for intelligent and quantitative appraising of skeletal maturity using cervical vertebras cone-beam CT images of Chinese girls [9]	2022	International Journal of Computer Assisted Radiology and Surgery	Xie Lizhe	PMC	English	methodology
Metaverse and Medical Diagnosis: A Blockchain-Based Digital Twinning Approach Based on MobileNetV2 Algorithm for Cervical Vertebral Maturation [10]	2023	Diagnostics	Moztarzadeh Omid	Scopus	English	Not assessing CVM
Knowledge distillation on individual vertebrae segmentation exploiting 3D U-Net [11]	2024	COMPUTERIZED MEDICAL IMAGING AND GRAPHICS	Serrador Luis	PMC	English	Not assessing CVM
Prediction of hand-wrist maturation stages based on cervical vertebrae images using artificial intelligence [12]	2021	Orthodontics and Craniofacial Research	Kim Dong Wook	Scopus	English	Not assessing CVM
Determination of growth and developmental stages in hand-wrist radiographs: Can fractal analysis in combination with artificial intelligence be used? [13]	2024	JOURNAL OF OROFACIAL ORTHOPEDICS-FORTSCHRITTE DER KIEFERORTHOPADIE	Gonca Merve	PMC	English	Not assessing CVM
Decision tree analysis for age estimation in living individuals: integrating cervical and dental radiographic evaluations within a South African population [14]	2023	International Journal of Legal Medicine	Uys A	Scopus	English	Not assessing CVM
Assessment of Skeletal Maturity in a Sample of the Saudi Population Using Cervical Vertebrae and Frontal Sinus Index: A Cephalometric Study Using Artificial Intelligence [15]	2023	CUREUS JOURNAL OF MEDICAL SCIENCE	Alfawzan Ahmed	PMC	English	studies not evaluating AI performance
The Fourth Cervical Vertebra Anterior and Posterior Body Height Projections (Vba) for the Assessment of Pubertal Growth Spurt [16]	2023	Applied Sciences	Camieriere Roberto et al.	PMC	English	studies not evaluating AI performance

Age estimation in Turkish children and young adolescents using fourth cervical vertebra [17]	2022	INTERNATIONAL JOURNAL OF LEGAL MEDICINE	Gulsahi Ayce et al.	PMC	English	studies not evaluating AI performance
iCVM: An Interpretable Deep Learning Model for CVM Assessment Under Label Uncertainty [18]	2022	IEEE Xplore	Liao Ni	PMC	English	studies not evaluating AI performance
Maturation of the middle phalanx of the third finger and cervical vertebrae: A comparative and diagnostic agreement study [19]	2014	Orthodontics and Craniofacial Research	Perinetti	PMC	English	studies not evaluating AI performance
A deep learning system for automated, multi-modality 2D segmentation of vertebral bodies and intervertebral discs [20]	2021	Bone	Suri Abhinav et al.	PMC	English	studies not evaluating AI performance

[1–20]

Refences:

1. Bajjad, A.A.; Gupta, S.; Agarwal, S.; Pawar, R.A.; Kothawade, M.U.; Singh, G. Use of Artificial Intelligence in Determination of Bone Age of the Healthy Individuals: A Scoping Review. *J World Fed Orthod* **2023**, doi:10.1016/j.ejwf.2023.10.001.
2. CAO, L.; HE, H.O.N.G.; HUA, F.A.N.G. CURRENT NEURAL NETWORKS DEMONSTRATE POTENTIAL IN AUTOMATED CERVICAL VERTEBRAL MATURATION STAGE CLASSIFICATION BASED ON LATERAL CEPHALOGRAMS. *Journal of Evidence-Based Dental Practice* 2024, *24*.
3. Rana, S.S.; Nath, B.; Chaudhari, P.K.; Vichare, S. Cervical Vertebral Maturation Assessment Using Various Machine Learning Techniques on Lateral Cephalogram: A Systematic Literature Review. *J Oral Biol Craniofac Res* **2023**, *13*, 642–651, doi:10.1016/j.jobcr.2023.08.005.
4. Mohammad-Rahimi, H.; Nadimi, M.; Rohban, M.H.; Shamsoddin, E.; Lee, V.Y.; Motamedian, S.R. Machine Learning and Orthodontics, Current Trends and the Future Opportunities: A Scoping Review. *American Journal of Orthodontics and Dentofacial Orthopedics* **2021**, *160*, 170-+, doi:10.1016/j.ajodo.2021.02.013.
5. Caloro, E.; Cè, M.; Gibelli, D.; Palamenghi, A.; Martinenghi, C.; Oliva, G.; Cellina, M. Artificial Intelligence (AI)-Based Systems for Automatic Skeletal Maturity Assessment through Bone and Teeth Analysis: A Revolution in the Radiological Workflow? *Applied Sciences* **2023**, *13*, 3860, doi:10.3390/app13063860.

6. Szemraj, A.; Wojtaszek-Słomińska, A.; Racka-Pilszak, B. Is the Cervical Vertebral Maturation (CVM) Method Effective Enough to Replace the Hand-Wrist Maturation (HWM) Method in Determining Skeletal Maturation?—A Systematic Review. *Eur J Radiol* 2018, *102*.
7. Goedmakers, C.; Pereboom, L.; Schoones, J.; de Leeuw den Bouter, M.; Remis, R.; Staring, M.; Vleggeert-Lankamp, C. Machine Learning for Image Analysis in the Cervical Spine: Systematic Review of the Available Models and Methods. *Brain Spine* **2022**, *2*, doi:10.1016/j.bas.2022.101666.
8. Kim, J.; Seo, H.; Park, S.; Lee, E.; Jeong, T.; Nam, O.H.; Choi, S.; Shin, J. Utilization of an Artificial Intelligence Program Using the Greulich-Pyle Method to Evaluate Bone Age in the Skeletal Maturation Stage. *THE JOURNAL OF THE KOREAN ACADEMY OF PEDTATRIC DENTISTRY* **2023**, *50*, doi:10.5933/jkapd.2023.50.1.89.
9. Xie, L.Z.; Tang, W.; Izadikhah, I.; Zhao, Z.Q.; Zhao, Y.; Li, H.; Yan, B. Development of a Multi-Stage Model for Intelligent and Quantitative Appraising of Skeletal Maturity Using Cervical Vertebras Cone-Beam CT Images of Chinese Girls. *Int J Comput Assist Radiol Surg* **2022**, *17*, 761–773, doi:10.1007/s11548-021-02550-7.
10. Moztarzadeh, O.; Jamshidi, M.; Sargolzaei, S.; Keikhaee, F.; Jamshidi, A.; Shadroo, S.; Hauer, L. Metaverse and Medical Diagnosis: A Blockchain-Based Digital Twinning Approach Based on MobileNetV2 Algorithm for Cervical Vertebral Maturation. *Diagnostics (Basel)* **2023**, *13*, doi:10.3390/diagnostics13081485.
11. Serrador, L.; Villani, F.P.; Moccia, S.; Santos, C.P. Knowledge Distillation on Individual Vertebrae Segmentation Exploiting 3D U-Net. *Computerized Medical Imaging and Graphics* **2024**, *113*, doi:10.1016/j.compmedimag.2024.102350.
12. Kim, D.W.; Kim, J.; Kim, T.; Kim, T.; Kim, Y.J.; Song, I.S.; Ahn, B.; Choo, J.; Lee, D.Y. Prediction of Hand-Wrist Maturation Stages Based on Cervical Vertebrae Images Using Artificial Intelligence. *Orthod Craniofac Res* **2021**, *24*, 68–75, doi:10.1111/ocr.12514.
13. Gonca, M.; Sert, M.F.; Gunacar, D.N.; Kose, T.E.; Beser, B. Determination of Growth and Developmental Stages in Hand–Wrist Radiographs. *Journal of Orofacial Orthopedics / Fortschritte der Kieferorthopädie* **2024**, doi:10.1007/s00056-023-00510-1.
14. Uys, A.; Steyn, M.; Botha, D. Decision Tree Analysis for Age Estimation in Living Individuals: Integrating Cervical and Dental Radiographic Evaluations within a South African Population. *Int J Legal Med* **2024**, doi:10.1007/s00414-023-03154-3.
15. Alfawzan, A.A. Assessment of Skeletal Maturity in a Sample of the Saudi Population Using Cervical Vertebrae and Frontal Sinus Index: A Cephalometric Study Using Artificial Intelligence. *Cureus Journal of Medical Science* **2023**, *15*, doi:10.7759/cureus.41811.
16. Cameriere, R.; Palacio, L.A. V; Nakas, E.; Galic, I.; Brkic, H.; Govorko, D.K.; Jerkovic, D.; Jara, L.; Ferrante, L. The Fourth Cervical Vertebra Anterior and Posterior Body Height Projections (*Vba*) for the Assessment of Pubertal Growth Spurt. *Applied Sciences-Basel* **2023**, *13*, doi:10.3390/app13031819.
17. Gulsahi, A.; Cehreli, S.B.; Galic, I.; Ferrante, L.; Cameriere, R. Age Estimation in Turkish Children and Young Adolescents Using Fourth Cervical Vertebra. *Int J Legal Med* **2020**, *134*, 1823–1829, doi:10.1007/s00414-020-02246-8.

18. Liao, N.; Dai, J.; Tang, Y.; Zhong, Q.; Mo, S. ICVM: An Interpretable Deep Learning Model for CVM Assessment Under Label Uncertainty. *IEEE J Biomed Health Inform* **2022**, *26*, 4325–4334, doi:10.1109/jbhi.2022.3179619.
19. Perinetti, G.; Perillo, L.; Franchi, L.; Di Lenarda, R.; Contardo, L. Maturation of the Middle Phalanx of the Third Finger and Cervical Vertebrae: A Comparative and Diagnostic Agreement Study. *Orthod Craniofac Res* **2014**, *17*, 270–279, doi:10.1111/ocr.12052.
20. Suri, A.; Jones, B.C.; Ng, G.; Anabaraonye, N.; Beyrer, P.; Domi, A.; Choi, G.; Tang, S.; Terry, A.; Leichner, T.; et al. A Deep Learning System for Automated, Multi-Modality 2D Segmentation of Vertebral Bodies and Intervertebral Discs. *Bone* **2021**, *149*, doi:10.1016/j.bone.2021.115972.
